# Supplementary material for: A high-resolution mRNA expression time course of embryonic development in zebrafish
Source: eLife. 2017 Nov 16;6:e30860. doi: 10.7554/eLife.30860 (PMC5690287; doi:10.7554/eLife.30860)
Supplement: Supplementary file 6. [file elife-30860-supp6.zip › biolayout-clusters-files/Cluster140-genes.html]

Cluster140


# Cluster140: Genes

| Ensembl ID | Gene Name | Chr | Start | End | Biotype |
| --- | --- | --- | --- | --- | --- |
| ENSDARG00000103691 | RFX7 (1 of many).1 | 25 | 365153 | 382834 | protein\_coding |
| ENSDARG00000102531 | anapc11 | 6 | 21773751 | 21779536 | protein\_coding |
| ENSDARG00000035122 | camsap1b | 5 | 64934177 | 64983881 | protein\_coding |
| ENSDARG00000093931 | fam101b | 15 | 25160767 | 25164414 | protein\_coding |
| ENSDARG00000062049 | hmha1b | 22 | 17586858 | 17627717 | protein\_coding |
| ENSDARG00000055917 | nipa1 | 6 | 37767825 | 37771817 | protein\_coding |
| ENSDARG00000045355 | tceb1a | 24 | 12839015 | 12844430 | protein\_coding |
| ENSDARG00000091198 | urm1 | 10 | 10354786 | 10367787 | protein\_coding |
| ENSDARG00000100916 | uvssa | 14 | 14764931 | 14849823 | protein\_coding |
